# Supplementary material for: Transcriptional Response of Circadian Clock Genes to an ‘Artificial Light at Night’ Pulse in the Cricket Gryllus bimaculatus
Source: Int J Mol Sci. 2022 Sep 26;23(19):11358. doi: 10.3390/ijms231911358 (PMC9570371; doi:10.3390/ijms231911358)
Supplement: Supplementary file 1 [file ijms-23-11358-s001.zip › Figure S1 Spectrogram.pdf]

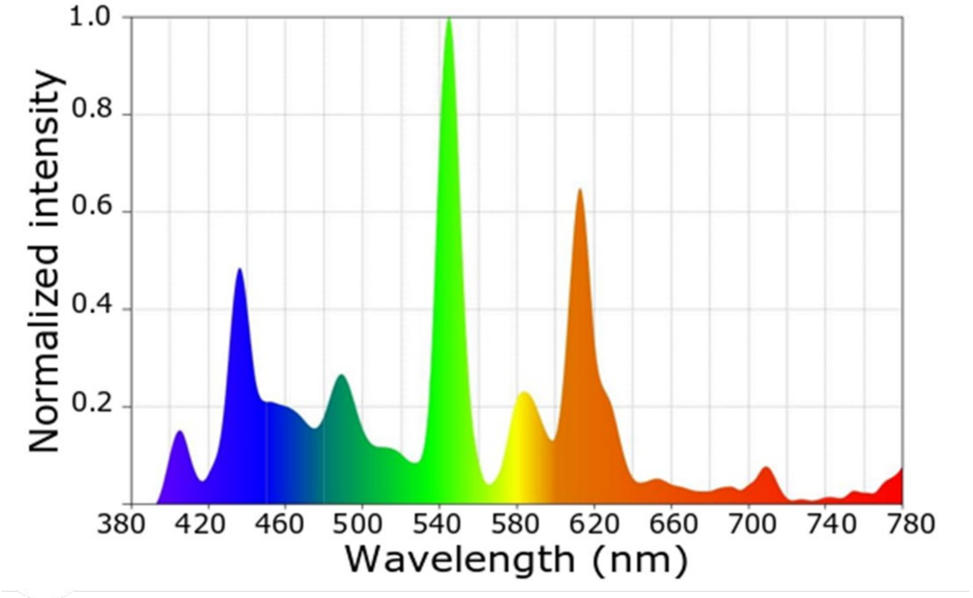

Figure S1. Spectrograms of the light bulb used for the acclimation and for the experiment. The light spectrum was recorded using the Sekonic Sprectromaster C-700 (North White Plans, NY, USA).
