# Supplementary material for: Transcriptional Response of Circadian Clock Genes to an ‘Artificial Light at Night’ Pulse in the Cricket Gryllus bimaculatus
Source: Int J Mol Sci. 2022 Sep 26;23(19):11358. doi: 10.3390/ijms231911358 (PMC9570371; doi:10.3390/ijms231911358)
Supplement: Supplementary file 1 [file ijms-23-11358-s001.zip › Table S1 - sample sizes.pdf]

Table S1. Sample sizes of individual male *Gryllus bimaculatus* adult crickets used for each tissue and treatment of the experiment. Each column represents a different gene.

Tissue	Treatment	*Rpl18a*	*actin*	*opLW*	*cry2*	*per*	*c-fosB*	*cry1*
**Brain**	0lx	9	9	8	9	8	9	8
	2lx	8	8	8	8	8	8	8
	5lx	6	6	6	6	6	6	6
	40lx	5	5	5	5	3	5	3
**Optic lobe**	0lx	4	4	4	3	3	4	2
	2lx	6	6	6	6	6	6	6
	5lx	6	6	6	6	6	6	6
	40lx	6	6	6	5	6	6	6
**Malpighian tubules**	0lx	14	14	13	14	12	13	12
	2lx	9	9	8	9	9	9	9
	5lx	12	12	10	11	11	11	11
	40lx	12	12	12	12	11	12	12
**Haemolymph**	0lx	5	5	4	5	4	4	3
	2lx	5	5	5	5	4	5	4
	5lx	5	5	4	4	4	4	4
	40lx	7	7	5	6	6	6	6
