# Supplementary material for: Transcriptional Response of Circadian Clock Genes to an ‘Artificial Light at Night’ Pulse in the Cricket Gryllus bimaculatus
Source: Int J Mol Sci. 2022 Sep 26;23(19):11358. doi: 10.3390/ijms231911358 (PMC9570371; doi:10.3390/ijms231911358)
Supplement: Supplementary file 1 [file ijms-23-11358-s001.zip › Table S2 - Primer sequence.pdf]

**Table S2.** Sequences of primers used for qPCR of circadian gene expression in adult male *Gryllus bimaculatus* crickets exposed to a pulse of one of four different ALAN intensities.

| Gene | Forward | Reverse |
|---|---|---|
| ***Rpl18a*** | GCTCCGGATTACATCGTTGC | GCCAAATGCCGAAGTTCTTG |
| ***actin*** | CCTGGCATTGCTGATAGGAT | CCTGCTTGGAGATCCACATT |
| ***opLW*** | CGCTCCTACATCCTCGTCTACTC | CGTTCATCTTCTTGGCTTGCT |
| ***cry2*** | TTCGACACGGAAGGTCTGCT | CGGCCAAATGATGCTACCC |
| ***per*** | GTGGAGGAGAACCGGTGTAA | CCAGGTTCCTCTTCAGCAAG |
| ***c-fosB*** | GTTGCCGTCTGAATGGAAAT | GGAGGTTTTGCACCACTTGT |
| ***cry1*** | GGGGCGAATACATCAAAAGA | CGACAATGAGGTGGAGGATT |
